# Point of care ultrasonography of quadriceps femoris muscle thickness for assessing nutritional status in critically ill children

**DOI:** 10.1038/s41598-025-01205-w

**Published:** 2025-05-15

**Authors:** Shereen A. Mohamed, Hanan M. Elsawy, Hafez M. Bazaraa, Mohamed H. Ghobashy, Mohamed Abdallah Abd El Megied

**Affiliations:** 1 Department of Pediatrics, Faculty of Medicine, Cairo University, Cairo, Egypt; 2 Department of Radiology, Faculty of Medicine, Cairo University, Cairo, Egypt

**Keywords:** Quadriceps, Ultrasonography, Critically ill, Mortality, Outcomes research, Paediatric research

## Abstract

Muscle wasting occurs early during critical illness. It is associated with poor PICU outcomes. Ultrasonography can detect muscle thickness in critically ill children. Study quadriceps muscle thickness to assess the nutritional status on admission and follow the muscle thickness change throughout the first week of admission in PICU using point-of-care ultrasonography. This cohort study was conducted on 55 critically ill mechanically ventilated children aged between 2 months and 14 years. Serial measurements of quadriceps muscle thickness were done by ultrasound, during the first week of admission. Quadriceps muscle thickness decreased by 16.4% over the 1st week of admission. The muscle wasting was significantly correlated with STRONGkids score, the inability to achieve target caloric requirements during the 1st week of admission, duration of ventilation, PRISM score, and mortality. Point of care ultrasonography is an easy tool for assessing PICU muscle wasting. Loss of muscle mass during the first week of PICU admission is correlated to mortality, thus it can be utilized in predicting PICU outcomes.

## Introduction

Critical illness is associated with metabolism, immunity, and endocrine system alterations, such as muscle catabolism and insulin resistance during the early phase of critical illness^1,2^. In critically ill adults, muscle wasting occurs due to low nutritional intake, immobilization, and catabolic hormones released during hospital stay^3^. Also, adult ICU patients requiring bed rest on admission had muscle wasting more obvious in the lower limb than in the upper limb^4^. In critically ill surgical adults, accurate muscle assessment by traditional examination is challenging in the ICU due to edema and hemodynamic instability^5^.

Quadriceps femoris muscle thickness changes occur on days 3 and 7 of admission compared to baseline thickness on the day of admission in the pediatric intensive care unit (PICU)^6^. The nutritional intake in critically ill children affects quadriceps femoris muscle thickness.^7^ This wasting is associated with longer PICU stays and poor outcomes^8–10^.

MRI is not suitable for serial muscle assessment in the PICU^11^. Fortunately, muscle ultrasonography can pick up muscle changes during PICU admission^12^. Regardless of the clinical case severity, ultrasonography could be done to avoid the hazards of ionizing radiation^13^. The quadriceps femoris muscle was chosen for muscle wasting assessment in preterm infants due to its large size and easy accessibility^14^. There is a lack of validated methods for muscle assessment in PICU^15^. And, there are a few studies on measuring muscle thickness using POCUS in critically ill children^15–18^.

This study was conducted in a developing country aiming to measure the quadriceps femoris muscle thickness in critically ill children on mechanical ventilation, using Point-of-Care Ultrasonography** (**POCUS) and explore the utility of this measurement to evaluate the nutritional status on admission and correlate it to nutritional intake and other factors during the first week of PICU stay. And investigate the correlation between muscle wasting and outcomes.

## Patients and methods

This cohort study was conducted in the PICU of Cairo University Children’s Hospital from January 2021 to August 2022. This 26-bed PICU manages general and post-surgical pediatric cases not including cardiac surgery (Renal replacement therapy is available) admitting children within the age range of 2 months to 14 years. Mechanically ventilated children were enrolled in this study on the day of admission. Sedatives were used but no muscle relaxants were utilized. Exclusion criteria included patients with muscular disease, absolute contraindication to enteral feeding as intestinal obstruction, on steroids in the past year on muscle relaxants and those admitted less than 3 days.

The Research Ethical Committee of the Faculty of Medicine, Cairo University approved the research protocol (code MD-34-2021) following the Helsinki Declaration 1964, as revised in 2000.

Before enrolment, informed written consent was obtained from the patient’s guardian.

A complete history was documented including age, sex, history of chronic diseases, operations, developmental pattern, type of feeding (breast or formula feeding), and use of any supplements before admission as a part of the assessment of nutritional status. Physical examinations including vital signs on admission (blood pressure, heart rate, and respiratory rate for age, saturation, and temperature) were accomplished.

Anthropometric measurements were plotted according to the guidelines of WHO growth charts. Head circumference was measured in children < 36 months. For children < 2 years, growth chats of weight for age and weight for length were used. For children > 2 years, weight for height and BMI were utilized. Mid-upper arm circumference (MUAC) was assessed in children up to 5 years old^8,19^.

Routine PICU investigations were performed on admission including random blood sugar level, CBC, CRP, serum electrolytes (Na, K, Mg), calcium profile (Ca, PO4, ALP), liver function tests, kidney function tests, and serum albumin. coagulation profile and cultures as blood, sputum, CSF, and urine if indicated and chest x-ray. Arterial blood gas is a part of investigations to confirm the indication of mechanical ventilation. Data collection included the diagnosis on admission, use of inotropes, need for dialysis, steroids or sedatives use during PICU stay, duration of mechanical ventilation, reintubation, and PICU length of stay.

Pediatric risk of Mortality Score (PRISM) was calculated in the first 12–24 h of admission. In 2015 a new update for PRISM improved its quality to predict mortality risk in PICU^20,21^. STRONGkids score was used to assess nutritional risk on admission^22^. Nutritional intake was calculated using the actual caloric and was compared to goal requirements guided by the Schofield equation^23^.

According to ASPEN guidelines, the goal for nutritional intake during the first week of admission is to reach at least two-thirds of the target requirements, beginning within 24–48 h and progressing in a stepwise manner. Enteral nutrition is the preferred method in the PICU, with a minimum protein intake of 1.5 g/kg/day. For critically ill children who are unable to receive enteral nutrition, parenteral nutrition should be initiated within the first week of admission^19^.

Extubating readiness in PICU is determined by PH > 7.25, Po2/Fio2 ≥ 150 or oxygen saturation is 90–95% on Fio2 ≤ 30% and positive end-expiratory pressure (PEEP) < 6, respiratory rate is set < 45, mean airway pressure is 7 and tidal volume is 4–5 ml/kg. Regarding clinical parameters, the patient should be vitally stable, and conscious with a gag reflex. The cause of respiratory failure is reversed with spontaneous breathing and hemoglobin level ≥ 7 mg/dl. The spontaneous breathing trial is switching the ventilator mode to CPAP, setting the pressure support to zero with the same PEEP level for 3 min then setting the pressure support to 5–8 cm H_2_O_2_ for 7 min. If any of the following parameters are present: bradycardia < 100/min more than 5 min, saturation < 85% with a 15% increase in FiO_2_ or increase in work of breathing, failure of spontaneous breathing trial is then documented^24^. Discharge criteria include hemodynamic stability and stable respiratory status with de-escalation of the level of care. Communication is essential with subspecialists if needed. A written summary and discharge plan should be deliberately delivered^25^.

On day 1 of admission, days 3, and 7 measurements of quadriceps femoris muscle thickness were done. Three readings were measured on each day and the average was recorded. Muscle ultrasonography was done with a linear probe, using a sufficient gel quantity to prevent image distortion^15^. The muscle thickness was measured in one transverse plane as repeated measurements in different planes are not commonly used in the literature^17^. The probe was placed perpendicular to the skin. The anatomical landmark was midway between the anterior superior iliac spine and the superior patellar edge^16^. The maximal pressure was applied to ensure accuracy and compress lower limb edema if present^5^. No discrepancy would be displayed if measurements were done midway between the landmarks or at 1/3 from the distal landmark^26^. The muscle thickness was the distance between the muscle superior border and the Femur cortex^15^. To minimize inter-rater variability, the radiologist (consultant of diagnostic and interventional radiology) performed the measurement each time using a GE ultrasound machine with a liner probe (11 L HZ). Since children’s muscle thickness varies by age group, the muscle wasting was computed as a percentage of change from the initial measurement.

### Statistical analysis

Data were analyzed using the IBM SPSS (Statistical Package for the Social Science; IBM Corp, Armonk, NY, USA) release 22 for Microsoft Windows ). The Kolmogorov–Smirnov test was used to assess normality for continuous variables. Descriptive analyses were performed to obtain the means, and deviations for quantitative data when normally distributed, and the median and IQR for skewed data. Also, numbers and frequencies for qualitative data were obtained.

Bivariate analyses were performed for normally distributed data using the independent sample t-test, paired student’s t-test, and one-way ANOVA test. Non-normally distributed variables were compared using the Mann–Whitney test (U test), Kruskal Wallis test, and the Chi-squared test for categorical variables.

Repeated measure ANOVA was used to assess the magnitude of change in the muscle thickness at each time point (day 1, 3, and 7) and to show whether loss of muscle thickness was greater during the early or late part of the PICU stay. Pearson and Spearman’s correlations were used to measure the interdependence between different quantitative variables and the change in muscle thickness. P-value < 0.05 was considered significant.

Univariate linear regression models were used to test for the test the association of each effector on the percent of change in muscle thickness (dependent variable). The entered effectors are the predictors of mortality. Forward and backward multivariate linear regression models were also performed to select the independent effectors on the percent of change in muscle thickness. We entered the same predictors of mortality. Two-sided* p* values less than 0.05 were considered statistically significant.

## Results

All patients admitted to the PICU at Cairo University Children’s Hospital from January 2021 to August 2022 were assessed, and 110 were found to fulfill the inclusion criteria. 55 patients’ guardians agreed to participate in this study. The cases had a median age of 14 (2–156) months, with 30 (54.5%) males and 25 (45.5%) females.

Some of our patients had more than one cause for PICU admission. Twenty-five (45.6%) of the study group were admitted for respiratory disorders, 21 (38.2%) had neurological disease, 9 (16.3%) sepsis, cardiac disorders comprised 5 (9.1%) of the study group, and other cases formed 10 (18.2%). History of chronic diseases, operations, and delayed developmental milestones were reported in 24 (43.6%), 6 (10.9%), and 14 (25.5%) cases respectively. Vitamins and minerals supplementation before admission were reported in about 17 cases (30.9%). Vitamin D, calcium, iron, folic acid, and L-carnitine were given to 52.9%, 29.4%, 29.4%, 17.6%, and 23.5% respectively. On admission, 67% of cases had normal random blood sugar levels, while hypoglycemia and hyperglycemia were reported in 22% and 11% of cases respectively. The most common metabolic disturbances revealed in arterial blood gases were respiratory alkalosis (38.5%) followed by metabolic acidosis, respiratory acidosis, and mixed acidosis in 27.3%, 18.2%, and 3.6% cases respectively. Sepsis screening in the form of cultures of various body fluids was performed. Positive results were obtained for blood, sputum, CSF, and urine specimens in 5 (9.1%), 25 (45.5%), 1 (1.8%), and 3 (5.5%) cases respectively. Sedatives were given to 42 (76.4%) cases with no muscle relaxants being utilized.

Using POCUS, the mean muscle thickness was 0.97 ± 0.40 (0.43–2.5) cm. The median age was 14 months which rationalizes the low mean muscle thickness. Within the first 24 h of admission, both PRISM and STRONGkids scores were calculated. The mean PRISM score was (13 ± 5 SD with a range of 3–24), while the mean STRONGkids score was (3 ± 1 SD with a range of 1–5) (Table 1).

**Table 1:** Assessment of the study group on admission.

	Mean ± SD	Min–max
Weight Z-score (median)	− 1.8 (− 2.6)	− 7.25 to 4.66
Height Z-score	− 0.55 ± 2.59	− 5.23 to 3.89
Mid-arm upper circumference Z-score	− 1.99 ± 2.27	− 7.65 to 1.68
Weight for length or BMI Z-score	− 1.9 ± 2.83	− 7.05 to 5.88
Head circumference Z-score	− 2 ± 3	− 7 to 5
Muscle measurement on admission (cm)	0.97 ± 0.40	0.43 to 2.5
PRISM score	13 ± 5	3 to 24
STRONGkids score	3 ± 1	1 to 5

SD: standard deviation; BMI: body mass index; cm: centimeter; PRISM score: Pediatric risk of Mortality Score; STRONGkids: Screening Tool for Risk on Nutritional Status and Growth.

Serial ultrasound measurements of muscle thickness were performed on the day of admission, day 3, and day 7. It decreased over the 1st week significantly (P < 0.001) (Table 2).

**Table 2:** The change in muscle thickness during the first week of admission.

	Muscle wasting percent	95% CI of the mean muscle thickness	P-value
1st day–3rd day	5.232	3.618–6.847	< 0.001
3rd day–7th day	5.873	4.158–7.587	
1st day–7th day	11.105	8.685–13.526	

Test of significance (Repeated measure ANOVA).

CI: Confidence interval.

34 (61.8%) of the cases failed to achieve at least two-thirds of the goal requirements by the end of the first week and significantly had muscle wasting (P < 0.001) (Table 3).

**Table 3:** Correlation of the muscle wasting percent and nutritional intake in the study group during the first week of admission.

	No (%)		Percent of change in muscle thickness	Test of significance	P-value
			Mean ± SD		
Achievement of goal requirements by the end of 1st week	Yes	21 (38.2%)	10.90** ± **9.61	3.741^#^	< 0.001
	No	34 (61.8%)	19.81** ± **7.88		

^#^: Independent sample t-test. *: ANOVA test, a: Spearman correlation, SD: standard deviation.

During the PICU stay, muscle wasting correlated significantly to PRISM and STRONGkids scores, older age, use of inotropes, and different diagnoses on admission (P = 0.002, 0.019, 0.001, 0.007, and < 0.001 respectively). However, there was no significant correlation between muscle wasting and other clinical data including sex, anthropometric measurements, vital signs on admission, history of chronic diseases, operations, developmental pattern, supplements used before admission, dialysis, sedatives, and steroid use during PICU admission (Table 4).

**Table 4:** Correlation of muscle wasting percent and clinical parameters.

		Correlation for muscle wasting percent	Test of significance	P- value
PRISM score		0.145b		0.002
STRONGkids score		0.315b		0.019
Age		0.421a		0.001
Weight Z-score		0.075a		0.589
Height Z-score		− 0.104b		0.448
MUAC Z-score		− 0.226		0.161
Weight for length or BMI Z-score		0.145a		0.29
Head circumference Z-score		− 0.147		0.33
		**Mean muscle wasting percentage ± SD**		
Sex	Female	18.72 ± 10.53	1.66^#^	0.102
	Male	14.48 ± 8.36		
Supplement use before admission	Yes	16.80 ± 11.16	0.230^#^	0.842
	No	16.23 ± 8.90		
Chronic diseases	Yes	19.19 ± 9.48	1.95^#^	0.056
	No	14.25 ± 9.18		
Operations	Yes	16.1 ± 7.27	1.35^#^	0.182
	No	16.45 ± 9.86		
Development	Normal	15.69 ± 9.71	0.953^#^	0.345
	Delayed	18.51 ± 9.09		
Sedation	Yes	16.23 ± 9.14	0.25^#^	0.804
	No	16.99 ± 11.16		
Inotropes	Yes	19.17 ± 8.58	2.78^#^	0.007
	No	12.26 ± 9.62		
Dialysis	Yes	22.78 ± 15.29	1.58^#^	0.119
	No	15.77 ± 8.77		
Steroid during admission	Yes	17.01 ± 9.83	− 0.92^#^	0.38
	No	13.71 ± 8.07		
Diagnosis				
Respiratory	Yes	13.6 ± 8.86	3.23^#^	< 0.001
Sepsis /infections	Yes	17.86 ± 7.28	3.92^#^	< 0.001
CNS causes	Yes	18.67 ± 10.81	3.98^#^	< 0.001
Cardiovascular	Yes	14.66 ± 7.93	7.93^#^	< 0.001
Blood pressure according to age	Low	17.69 ± 8.1	2.02*	0.078
	Normal	14.35 ± 9.24		
	High	27.57 ± 16.42		
Heart rate according to age	Normal	17.41 ± 10.55	1.35^#^	0.182
	High	16.13 ± 9.37		

^#^: Independent sample t-test. *: ANOVA test a: Spearman correlation b: Pearson correlation.

PRISM score: Pediatric risk of Mortality Score; STRONGkids: Screening Tool for Risk on Nutritional Status and Growth; MUAC: Mid-arm upper circumference; BMI: body mass index; SD: standard deviation; CNS: central nervous system.

The percentage of muscle wasting was significantly correlated to the low WBC count, low PLT count, prolonged PTT level, positive sputum culture, elevated CRP, and serum creatinine (P = 0.021, 0.018, 0.011, 0.045, 0.047, and 0.008respectively). No significant correlation was found between muscle wasting and other investigations (Table 5).

**Table 5:** Relation between muscle wasting percent and investigations of the study group.

		Muscle wasting percent Mean ± SD	Test of significance	P-value
Random blood sugar	Low	15.58 ± 7.62	0.132*	0.87
	Normal	16.87 ± 10.26		
	High	15.20 ± 9.75		
Acid–base status	Normal	17.78 ± 12.85	0.481*	0.75
	Metabolic acidosis	15.76 ± 8.64		
	Respiratory acidosis	16.67 ± 9.34		
	Respiratory alkalosis	15.48 ± 9.78		
	Mixed acidosis	24.90 ± 2.96		
Blood culture	No growth	16.13 ± 9.85	0.672^#^	0.505
	Positive	19.16 ± 5.67		
Sputum culture	No growth	13.38 ± 9.47	3.621*	0.045
	Inhibited growth	14.29 ± 4.83		
	Positive	19.86 ± 9.43		
CXR	Normal	18.30 ± 9.48	1.57^#^	0.122
	Pneumonia	14.30 ± 9.36		
	**Correlations with percent of muscle wasting**			**P-value**
WBCs	− 0.312b			0.021
PLTs	− 0.318b			0.018
PTT	0.340b			0.011
CRP	0.269b			0.047
Creatinine	0.353a			0.008

^#^: Independent sample t-test. *: ANOVA test a: Spearman correlation b: Pearson correlation.

SD: standard deviation; CXR: chest x-ray; WBCs: White blood cells; PLTs: Platelets; PTT: Partial thromboplastin time; CRP: C-reactive protein.

The mean duration of mechanical ventilation was 13 ± 13 (1–70) days, while the mean duration of PICU admission was 17 ± 14 (7–76) days. 33 (60%) children died. Muscle wasting was significantly correlated with mortality risk and duration of mechanical ventilation (P < 0.001 and 0.036 respectively). However, there was no correlation between muscle wasting and reintubation events or PICU length of stay (Table 6).

**Table 6:** Relation between the outcome of the study group and muscle wasting percent over the first week of admission.

Outcome		N (%)	Mean of muscle wasting percent ± SD	Test of significance (t-test)	P-value
Reintubation	Yes	14 (25.5%)	20.05 ± 7.74	2.81^#^	0.09
	No	41(74.5%)	15.17 ± 9.87		
Mortality risk	Died	33 (60%)	20.24 ± 8.18	4.1^#^	< 0.001
	Discharged	22 (40%)	10.7 ± 8.86		
	**Mean ± SD**	**Correlation for muscle wasting percent**		**P-value**	
Mechanical ventilation duration (days)	13 ± 13	0.283*		0.036	
PICU length of stay (days)	17 ± 14	0.144*		0.295	

^#^: Independent sample t-test *: Spearman correlation, SD: standard deviation; PICU: pediatric intensive care unit.

Table 7 showed that the history of chronic diseases, operations, and developmental delay was significantly associated with mortality risk (P = 0.011, 0.034, and 0.005 respectively). Also, the presence of respiratory illness on admission, positive sputum culture, inotropic use, hypotension, duration on mechanical ventilation, low serum albumin, and prolonged PTT levels were significantly associated with mortality risk (P = 0.02, 0.032, < 0.001, 0.027, 0.001, 0.006 and 0.007 respectively). The inability to achieve goal nutritional requirements and STRONGkids scores were significantly associated with mortality risk (P < 0.001). The Muscle thickness on days 3 and 7 was significantly associated with mortality (P = 0.048 and 0.009 respectively).

**Table 7:** Mortality predictors in the study group.

		Outcome		Chi-square test	P -value
		Died N (%)	Discharged N (%)		
Chronic diseases	No	14 (45.1)	17 (54.8)	6.518	0.011
	Yes	19 (79.2)	5 (20.8)		
Operations	No	27 (55.1)	22 (44.9)	4.49	0.034
	Yes	6 (100%)	0 (0%)		
Development	Delayed	13 (92.9%)	1 (7.1%)	8.015	0.005
	Normal	20 (48.80%)	21 (51.2%)		
Inotropes	No	5 (22.7%)	17 (77.3%)	20.11	< 0.001
	Yes	28 (84.8%)	5 (15.2%)		
Respiratory illness	No	23 (70%)	7 (31.2%)	5.18	0.02
	Yes	10 (30%)	15 (68.81%)		
Blood pressure	Low	18 (81.8%)	4 (18.2%)	7.244	0.027
	Normal	13 (43.3%)	17 (56.7%)		
	High	2 (66.7%)	1 (33.3%)		
Sputum culture	No growth	10 (40%)	15 (60%)	6.901	0.032
	Inhibited growth	4 (80%)	1 (20%)		
	Positive	19 (76%)	6 (24%)		
Achievement of goal requirements in the 1st week	No	29 (85.3%)	5 (14.7%)	25.58	< 0.001
	Yes	4 (19%)	17 (81%)		
		**Died: 33 (60%)**	**Discharged: 22 (40%)**	**t-test**	
		**Mean ± SD**			
PRISM score		15.06 ± 3.88	9 ± 3.53	6	< 0.001
STRONGkids score		3.03 ± 1.04	1.95 ± 0.85	4.35	< 0.001
Albumin		3.08 ± 0.61	3.85 ± 0.65	-2.856-	0.006
PTT		47.42 ± 22.95	32.6 ± 10.89	2.83	0.007
Mechanical ventilation duration		18 ± 14.46	6.18 ± 6.13	3.55	0.001
Muscle thickness on day 3		0.81 ± 0.32	1.3 ± 0.41	-2.023-	0.048
Muscle thickness on day 7		0.72 ± 0.26	0.99 ± 0.31	-2.727-	0.009

SD: standard deviation; PRISM score: Pediatric risk of Mortality Score; STRONGkids: Screening Tool for Risk on Nutritional Status and Growth; PTT: Partial thromboplastin time.

Univariate analysis revealed that six factors significantly affected the percent of change in muscle thickness (inotropic use, positive sputum culture, achievement of nutritional requirement in the first week, duration on mechanical ventilation, PRISM score, and STRONGkids score) (Table 8). Forward analysis revealed that achievement of goal requirements in the 1st week and sputum culture are the only independent effectors, while backward analysis concluded that added PRISM score and calories as independent effectors on the percent of change in muscle thickness (Tables 9 and 10).

**Table 8:** Univariate regression analysis of the association between the percent of change in muscle thickness and different predictors of mortality.

Variables	Unstandardized Coefficients		95% CI		t-test	P-value
	B	SE	Lower	Upper		
Respiratory	4.945	2.521	− 0.111	10.002	1.962	0.055
(Constant)	− 18.653	1.7	− 22.062	− 15.224	− 10.975	0.000
Chronic diseases	− 4.939	2.532	− 10.018	0.139	− 1.951	0.056
(Constant)	− 14.250	1.673	− 17.604	− 10.895	− 8.520	0.000
Operations	0.341	4.170	− 8.022	8.704	0.082	0.935
(Constant)	− 16.442	1.377	− 19.205	− 13.680	− 11.939	0.000
Development	− 2.822	2.959	− 8.757	3.113	− 0.954	0.345
(Constant)	− 15.687	1.493	− 18.681	− 12.692	− 10.508	0.000
Inotropes	− 6.910	2.478	− 11.880	− 1.940	− 2.789	0.007
(Constant)	− 12.259	1.919	− 16.109	− 8.409	− 6.387	0.000
Achievement of requirements in the 1st week	8.905	2.380	4.131	13.678	3.742	0.000
(Constant)	− 19.805	1.471	− 22.754	− 16.855	− 13.468	0.000
Sputum culture	− 6.323	2.462	− 11.261	− 1.385	− 2.568	0.013
(Constant)	− 13.531	1.660	− 16.860	− 10.202	− 8.152	0.000
PRISM score	− 0.835	0.251	− 1.338	− 0.331	− 3.324	0.002
(Constant)	− 5.918	3.370	− 12.677	0.840	− 1.756	0.085
STRONGKIDS score	− 2.774	1.148	− 5.076	− 0.471	− 2.416	0.019
(Constant)	− 9.295	3.191	− 15.695	− 2.894	− 2.913	0.005
Calories (kcal)	0.005	0.003	− 0.002	0.011	1.461	0.150
(Constant)	− 18.886	2.123	− 23.143	− 14.628	− 8.897	0.000
MV duration (days)	− 0.206	0.096	− 0.398	− 0.014	− 2.151	0.036
(Constant)	− 13.684	1.776	− 17.247	− 10.122	− 7.705	0.000

B: Beta Coefficient; SE: standard error; CI: Confidence interval; PRISM score: Pediatric risk of Mortality Score; STRONGkids: Screening Tool for Risk on Nutritional Status and Growth; MV: mechanical ventilation.

**Table 9:** Forward multivariate linear regression analysis of the association between the percent of change in muscle thickness and different predictors of mortality.

Model		Unstandardized Coefficients		95% CI		t-test	P-value
		B	SE	Lower	Upper		
	Achievement of requirements in the 1st week	8.263	2.295	3.659	12.868	3.601	0.001
	Sputum culture	− 5.386	2.239	− 9.879	− 0.894	− 2.406	0.020

B: Beta coefficient; SE: standard error; CI: Confidence interval.

**Table 10:** Backward multivariate linear regression analysis of the association between the percent of change in muscle thickness and different predictors of mortality.

Model		Unstandardized coefficients		95% CI		t-test	P-value
		B	SE	Lower	Upper		
	PRISM score	− 0.531	0.253	− 1.039	− 0.024	− 2.104	0.040
	Sputum culture	− 4.910	2.137	− 9.202	− 0.619	− 2.298	0.026
	Calories (kcal)	− 0.008	0.004	− 0.016	− 0.001	− 2.148	0.037
	Achievement of requirements in the 1st week	11.083	3.269	4.517	17.648	3.390	0.001

B: Beta coefficient; SE: standard error; CI: Confidence interval; PRISM score: Pediatric risk of Mortality Score.

**Fig. 1:**
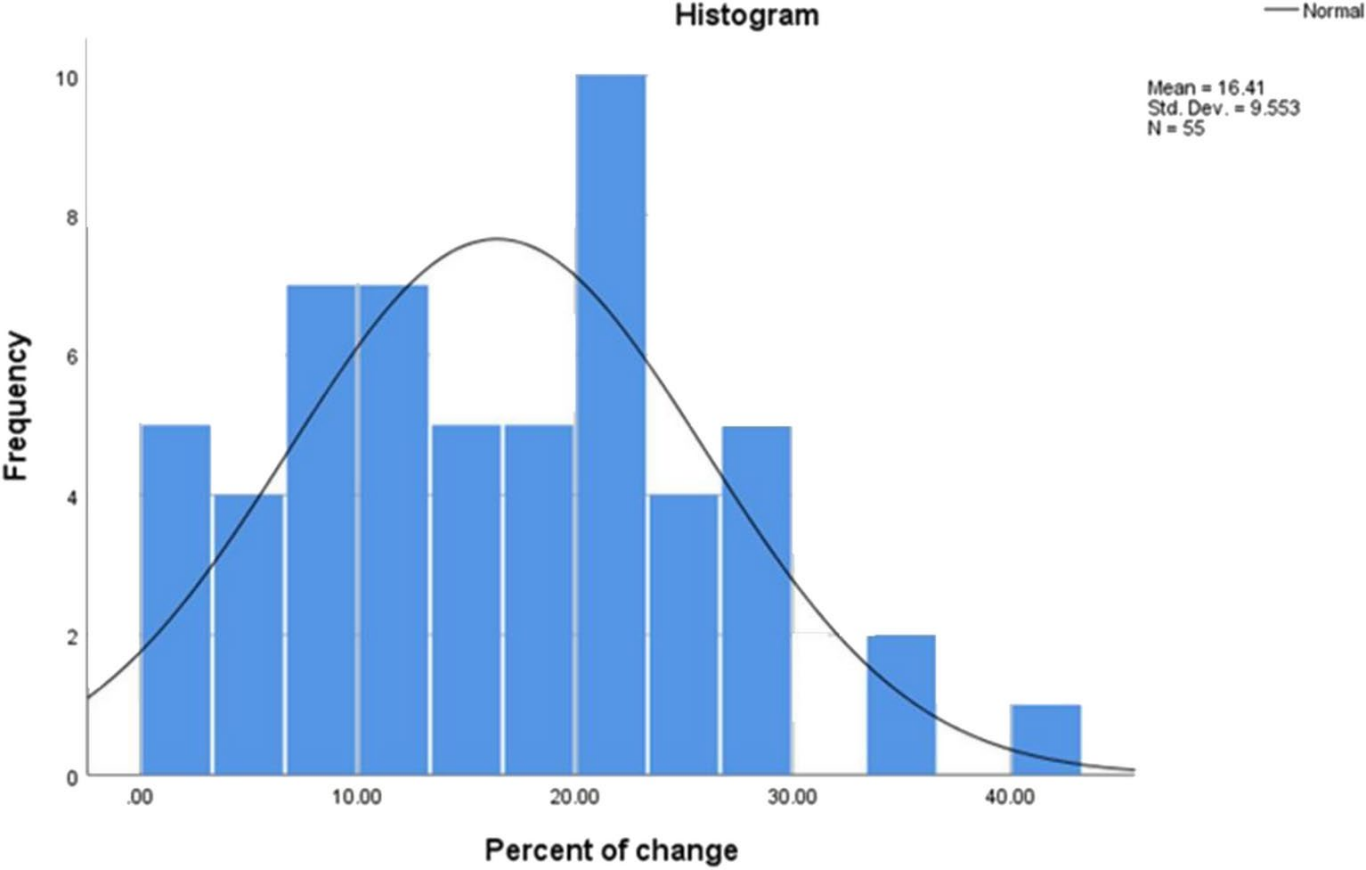
Histogram of the percentage of muscle wasting over the first week of admission.

The percentage of muscle wasting was picked up during serial ultrasound assessment of muscle thickness during the first week of PICU admission. The mean percentage of muscle wasting was 16.41% over the first week of admission (Fig. 1).

**Fig. 2:**
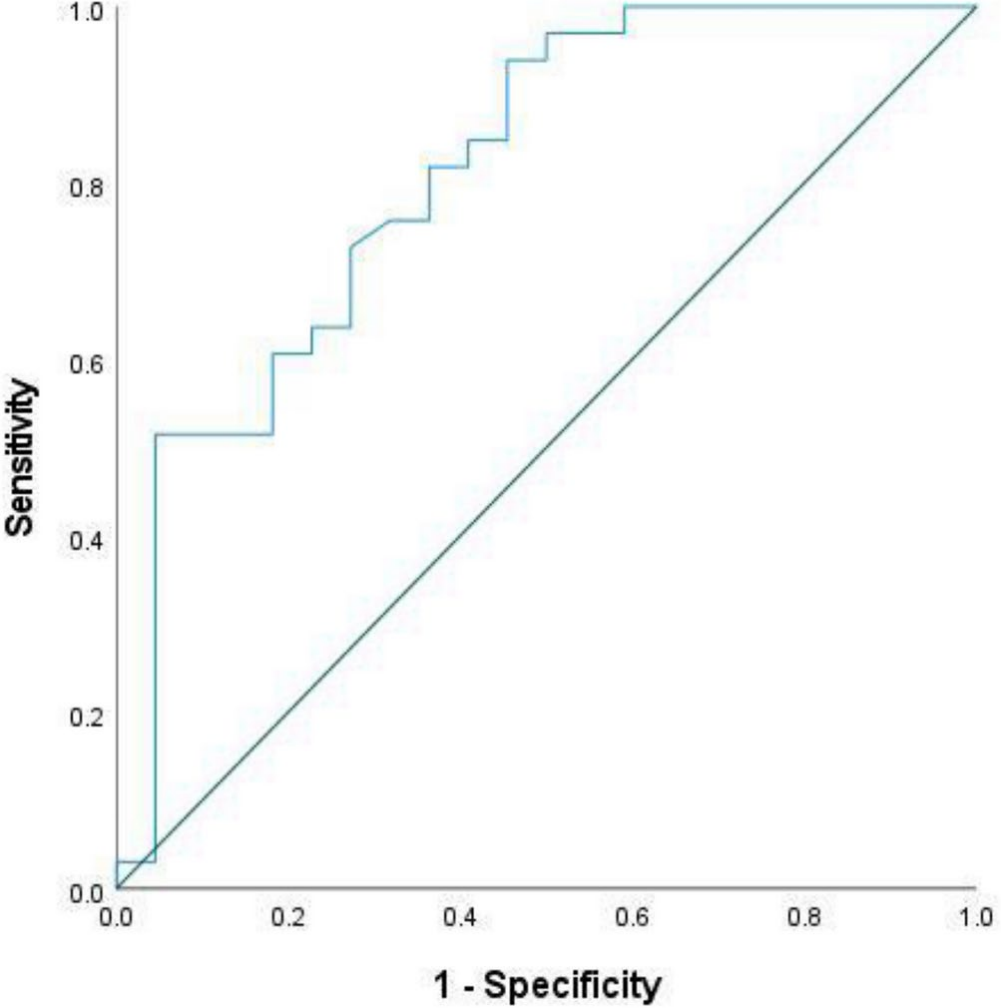
ROC curve for discrimination between survivors and non-survivors using the percent of muscle wasting.

The sensitivity and specificity of the percent of muscle wasting in discrimination between survivors and non-survivors were 100% and 41% respectively at a cutoff of 6.34% of muscle wasting. The Receiver-operating characteristic (ROC) curve showed that the percent of muscle wasting measurement is a significant tool in discrimination between survivors and non-survivors (AUC 81.2%, P < 0.001) (Fig. 2).

## Discussion

In critically ill children, malnutrition increases the risk of mortality and prolongs PICU stay. It can be assessed by evaluating muscle wasting.^9,27,28^. A practical method for estimating muscle wasting during a PICU stay is measuring muscle thickness using POCUS^13,29^. During serial measurements of the quadriceps femoris muscle in this study, there was a mean loss in muscle thickness of 16.4% during the 1st week of admission. Several studies conducted on different criteria of the studied groups, both in adult and pediatric populations, have used ultrasound to monitor quadriceps muscle thickness. (11,13,16,17,27,28). In pediatrics, De Figueiredo et al. study included critically ill children aged between 28 days and 14 years whether they were ventilated or not. The US assessment noted a significant percentage (14%) of quadriceps muscle thickness loss over the first week of admission^12^. Likewise, Valla et al. reported a nearly 10% decrease in quadriceps muscle thickness on day 5 in PICU in mechanically ventilated children aged 15 years or younger whether they were fully sedated or cooperative during measurements by ultrasound^17^. Also, Johnson et al. observed that muscle thickness reduced by 1.5% per day within 5 to 7 days of admission in mechanically ventilated children^16^.

Subtle muscle contraction, even in sedated critically ill children may temporarily preserve muscle thickness during the early days of admission.^17^. Though 76.4% of our patients were sedated, they developed muscle wasting. In contrast to our study, Salinas et al. objected to quadriceps muscle wasting in the first 4 days of PICU admission^30^. Whereas, another study noticed that quadriceps muscle thickness was stable during the PICU stay^31^.

Critically ill children are in a catabolic state and it is a great challenge to achieve the targeted caloric intake for PICU patients^32^. 61.8% of our patients didn’t achieve minimal nutritional intake in the first week. Both added significant quadriceps muscle wasting. This was in line with other studies in critically ill children that found muscle wasting was associated with deficient caloric intake. They found that for each decrease in the goal of energy by 1%, the quadriceps muscle size declined by 0.14% and 0.22% respectively during PICU admission^7,33^. In contrast, two studies found no association between caloric intake and quadriceps muscle thickness^12,17^.

Malnutrition is a risk factor for muscle wasting in critically ill children^28^. Several tools were validated to assess nutritional status, even if lengthy and consisting of numerous parameters, we applied the STRONGkids score and anthropometric measurements to identify malnutrition on the day of admission. This study group scored from 1 to 5. Similarly, Aziz et al. applied the STRONGkids score which ranged from 0 to 5^34^. In our study, the STRONGkids score was correlated with muscle wasting. Similar findings were reported in other research undertaken in the PICU^10^. Alternatively, a correlation between the NUTRIC score (to measure nutritional status in critically ill adults) and quadriceps muscle wasting was affirmed^35^.

As our patients presented with a high PRISM score, they encountered a high mortality rate (60%).

Also, an increased PRISM score was associated with quadriceps muscle wasting. Likewise, the STRONGkids score was correlated to mortality and muscle wasting. A systematic and meta-analysis review perceived that under-nourished cases had a higher risk of PICU mortality^36^. Field-Ridley et al. found that muscle weakness was also related to elevated mortality risk in PICU^10^. Individual and prompt nutritional support is recommended as malnourishment increases muscle breakdown and the risk of mortality in PICU^37^.

The duration of PICU stay had no significant correlation with muscle wasting or mortality risk in our study as reported by others^12,38^. Opposingly, Field-Ridley et al. found that muscle weakness in PICU was associated with prolonged PICU length of stay^10^. Nevertheless, the duration of mechanical ventilation was correlated with increased quadriceps muscle wasting and mortality risk in our cases. This was in line with Field-Ridley et al.^10^.

In our study, we observed the duration of mechanical ventilation, prolonged PICU length of stay, and the inability to achieve goal nutritional requirements mounted the risk of mortality. Similar findings were observed in several studies in developing countries^39^^40^. It is advocated that adequate caloric intake during PICU admission significantly reduces the duration of mechanical ventilation and hospital stay^19,27,41^.

Limitations of the current study; First, we didn’t measure the cross-sectional area or echogenicity of the quadriceps femoris muscle, however, Jain et al. found that quadriceps muscle echogenicity changed in critically ill children by 16% over the 1st week of PICU admission with no significant change in quadriceps muscle thickness^31^. Second, we didn’t consider or assess the effect of the cumulative fluid balance on muscle thickness, since moderate fluid imbalance had no significant impact on muscle thickness^42^. Third, our study did not assess reproducibility, intra-observer reliability, and inter-observer reliability using ultrasound as the radiologist measured each time.

## Conclusion and recommendations

During the first week of PICU admission, POCUS exposed a considerable loss of quadriceps muscle thickness, which was linked to insufficient nutritional intake. Muscle wasting was correlated to increased mortality risk and the duration of mechanical ventilation. We emphasize the importance of prompt achievement of nutritional requirements, in conjunction with serial quadriceps muscle thickness measurement using POCUS to assess nutritional status.

## Data Availability

The datasets used and analyzed during the current study are available from the corresponding author upon reasonable request.
